# Exploring the relationships between clinical manifestations and sex hormones, thyroid hormones, and menstrual factors in female Parkinson’s disease patients

**DOI:** 10.3389/fmed.2025.1573768

**Published:** 2025-09-24

**Authors:** Fan Wang, Jing Zhao, Jichen Du, Jilai Li, Peifu Wang, Zhong Yi, Tao Feng, Zhirong Wan

**Affiliations:** ^1^Department of Neurology, Aerospace Center Hospital, Beijing, China; ^2^The First Department of Geriatrics, Aerospace Central Hospital, Beijing, China; ^3^Center for Movement Disorders, Department of Neurology, Beijing Tiantan Hospital, Capital Medical University, Beijing, China

**Keywords:** female Parkinson’s disease patients, motor complications, sex hormones, thyroid hormones, menstrual factors

## Abstract

**Background:**

Parkinson’s disease (PD) affects both sexes, but there are notable differences in its clinical manifestations and management in women.

**Objective:**

This study aimed to compare variations in sex and thyroid hormone levels and menstrual factors between postmenopausal women with and without motor complications (PWP-MC and PWP-nMC, respectively) and analyze their correlations with motor complications.

**Methods:**

Ninety-five Postmenopausal Women with Parkinson’s Disease (PWP) provided data on age at menarche, age at menopause, menstrual cycle duration (interval between cycle starts (days)), total years of menstruation (menopausal age - age at menarche), thyroid disease history, and gynecological surgical history. Six sex hormones and seven thyroid function indicators were measured, followed by an analysis of the relationships among sex hormone levels, thyroid function, menstrual factors, clinical characteristics, and disease severity in PWP. The effects of sex hormones and menstrual factors on motor complications in PWP were also investigated.

**Results:**

The results revealed several key findings: (1) PWP-MC exhibited lower serum prolactin levels than PWP-nMC (*p* < 0.05). (2) In PWP, serum estradiol levels were negatively correlated with Hamilton Anxiety Rating Scale (HAMA) scores (*r* = −0.208, *p* = 0.043). (3) There were no statistically significant differences in age at menarche, age at menopause, menstrual cycle duration, menstruation duration (Days of active bleeding per cycle), or total years of menstruation between PWP-MC and PWP-nMC (*p* > 0.05). (4) In PWP, age at menarche was negatively correlated with Mini-Mental State Examination (MMSE) scale scores (*r* = −0.264, *p* = 0.01) and Montreal Cognitive Assessment (MoCA) scale scores (*r* = −0.297, *p* = 0.004); total years of menstruation were positively correlated with MoCA scale scores (*r* = 0.278, *p* = 0.006); menstrual cycle duration was negatively correlated with Unified Parkinson’s Disease Rating Scale, Part III (UPDRS-III) scores (*r* = −0.246, *p* = 0.016) and Hoehn–Yahr (H-Y) stages (*r* = −0.236, *p* = 0.021); and menstruation duration was positively correlated with HAMA (*r* = 0.215, *p* = 0.036) and Non-Motor Symptoms Scale (NMSS) scores (*r* = 0.214, *p* = 0.037). (5) In PWP-MC, age at menopause and total years of menstruation were positively correlated with Hamilton Depression Rating Scale (HAMD) scores (*r* = 0.335, *p* = 0.043; *r* = 0.352, *p* = 0.033, respectively); menstruation duration was negatively correlated with UPDRS-III scores (*r* = −0.362, *p* = 0.028) and positively correlated with HAMD (*r* = 0.329, *p* = 0.047) and HAMA (*r* = 0.451, *p* = 0.005) scores; and menstruation duration was positively correlated with NMSS (*r* = 0.325, *p* = 0.050) scores. (6) In PWP-nMC, age at menarche was negatively correlated with MMSE (*r* = −0.332, *p* = 0.011) and MoCA (*r* = −0.296, *p* = 0.024) scores; total years of menstruation were negatively correlated with UPDRS-III (*r* = −0.287, *p* = 0.029) scores and positively correlated with MMSE (*r* = 0.316, *p* = 0.016) and MoCA (*r* = 0.337, *p* = 0.010) scores. (7) Compared with PWP-nMC, PWP-MC had lower serum triiodothyronine levels (*p* < 0.05) and higher serum thyroid-stimulating hormone levels (*p* < 0.05). (8) In PWP-MC, triiodothyronine levels were negatively correlated with UPDRS-III scores (*r* = −0.344, *p* = 0.037) and H-Y stages (*r* = −0.445, *p* = 0.005); free triiodothyronine (FT3) levels were negatively correlated with H-Y stages (*r* = −0.476, *p* = 0.003); and free thyroxine (FT4) levels were negatively correlated with UPDRS-III scores (*r* = −0.422, *p* = 0.009) and H-Y stages (*r* = −0.365, *p* = 0.026).

**Conclusion:**

These findings suggest that the occurrence of motor complications in PWP may be correlated with prolactin, T3, and FT3 levels. Additionally, attention should be given to thyroid function and serum T3, T4, FT3, and FT4 levels in PWP, as lower levels may be associated with more severe motor symptoms, higher H-Y stages, and poorer cognitive function. Furthermore, older age at onset was inversely associated with motor complications in PWP, whereas a longer disease duration and higher NMSS score are risk factors.

## 1 Introduction

Parkinson’s disease (PD) is the second most common neurodegenerative disorder and is characterized by motor impairments, tremors, and muscle rigidity. PD primarily results from the degeneration of dopaminergic neurons in the substantia nigra, leading to a decrease in dopamine levels. PD is prevalent worldwide, with an estimated 6 million people affected globally (1, 2).

However, along with these primary motor symptoms, PD can also give rise to a range of secondary motor complications that significantly impact the quality of life of affected individuals. Two prominent motor complications in PD patients are symptom fluctuations and dyskinesia (3). These complications are related to medication use and disease progression. Levodopa (LD) is one of the primary drugs used to treat PD, but its long-term use can trigger the occurrence of or increase motor complications. Studies have shown that the incidence of motor complications can be as high as 50% in patients who have been taking LD for more than 5 years, reaching 100% in patients who develop the disease at a young age and affecting a greater proportion of females (4). Various factors, including uncontrollable and controllable factors, influence the fluctuation of PD symptoms. Uncontrollable factors include gender, age of onset, ethnicity, disease duration, rate of disease progression, PD subtype, disease severity, genetic polymorphisms, etc. Controllable factors mainly include the use of therapeutic drugs, such as the timing of LD treatment and LD dosage (5–7). Currently, the high-risk factors believed to induce LD-induced dyskinesia (LID) include female sex, low body weight, a long disease duration, large single LD doses, a high LD equivalent daily dose (LEDD), etc (8). Warren et al. reported that LID is closely related to LD dosage, especially in female patients. This relationship is particularly evident when the LD daily dosage is ≥400 mg/d (9). Understanding the clinical characteristics and potential mechanisms of these motor complications has helped in the development of effective interventions. Further research is needed to explore personalized approaches and novel treatment options to improve the management of PD and enhance the quality of life of patients with PD.

Research suggests that the prevalence of PD is slightly greater in men than in women, with a male-to-female ratio of approximately 1.5:1 (10). However, studies have shown that female PD patients may have different motor and non-motor symptoms than their male counterparts do (4). For example, female PD patients tend to have a greater prevalence of the tremor-dominant subtype and less severe motor symptoms (4). Additionally, female patients may have a greater risk of developing dyskinesias and experiencing fluctuations in their medication response (4). In addition to motor symptoms, female PD patients often experience non-motor manifestations. For example, they may be more prone to depression, anxiety, and cognitive impairment (11, 12). Hormonal factors, such as menopausal status and estrogen levels, have been suggested to contribute to these differences (13). Koller et al. first proposed the relationship between estrogen and movement disorders (14). Sex hormones play an essential role in the process of PD development, as estrogen secretion levels can affect dopamine function in the substantia nigra and striatum, as well as a role in the therapeutic effects of dopamine medications (15). The neuroprotective effect of estrogen is one of the reasons for the lower incidence of PD in the female population (16). Low estrogen levels are positively correlated with PD, and estrogen replacement therapy after menopause can reduce the incidence of PD in women and delay the age at PD onset (17). Clinical studies have also revealed that some premenopausal women with PD (PWP) often experience exacerbated motor and non-motor symptoms and a decrease in the efficacy of LD during the premenstrual and menstrual phases when estrogen levels are at their lowest (18). There is a specific association between thyroid function and the pathogenesis of PD (19–22). Subclinical hyperthyroidism is more common in PD patients than in individuals without PD, and the levels of free thyroxine (FT4) are elevated in newly diagnosed, untreated PD patients (23). Research has indicated that thyroid hormone levels are closely related to motor symptoms in PD patients (19, 24). These findings underscore the importance of addressing not only motor symptoms but also the broader range of challenges faced by female PD patients.

In conclusion, while PD affects both sexes, there are essential differences in the clinical presentation and management of the disease in women. Understanding these unique characteristics is critical for healthcare providers to provide personalized care and optimize treatment outcomes for female PD patients.

## 2 Materials and methods

### 2.1 Subjects

This study included 95 postmenopausal female Chinese PD patients admitted to PD clinics at Beijing Tiantan Hospital and Aerospace Center Hospital from September 2019 to June 2021. The inclusion criteria for patients were as follows: (1) met the diagnostic criteria for PD in China (2016 criteria); (2) were postmenopausal females. The exclusion criteria included the following: (1) the presence of intracranial organic diseases; (2) the presence of Parkinson’s plus syndrome (multiple system atrophy, progressive supranuclear palsy, Lewy body dementia, etc.); (3) the presence of secondary Parkinson’s syndrome (vascular, toxic, traumatic, metabolic, etc.); (4) the presence of significant mental disorders or severe organic diseases; (5) the presence of consciousness disorders and current use of thyroid hormones, estrogen, or progesterone supplements; (6) the inability to cooperate with scale assessment for various reasons; and (7) Non-female patients. The Medical Ethics Committees of the Aerospace Center Hospital and Beijing Tiantan Hospital approved this study. All participants in the study signed informed consent forms. The ethical approval number is 20190614-SF-06.

### 2.2 Collection of demographic data

The demographic data collected included age, height, weight, body mass index (BMI), age at menarche, age at menopause, menstrual cycle duration, menstrual phase, total duration of menstruation (age at menopause - age at menarche), history of thyroid disease, and history of thyroid hormone, estrogen, or progesterone supplement use. Data were collected through face-to-face questionnaires or electronic systems in hospital outpatient departments. The height measurements were precise to 1 cm, and the weight measurements were precise to 0.1 kg. Patients with a BMI < 20 kg/m2 were classified into the PD-low-BMI group, whereas those with a BMI ≥ 20 kg/m2 was classified into the PD-non-low-BMI group.

### 2.3 Clinical data collection

The clinical data collected included age at onset, duration of PD, initial symptoms, selection of initial medication, current LEDD, and other clinical information.

### 2.4 Scale assessment

(1) The Unified Parkinson’s Disease Rating Scale, Part III (UPDRS-III), was used to assess motor function (25), and the evaluation was conducted during the “off period”. (2) The Unified Parkinson’s Disease Rating Scale, Part IV (UPDRS-IV), was used to assess dyskinesia, and the evaluation was conducted during the “on period”. (3) The Hoehn-Yahr (H-Y) staging scale was used to assess the severity of the disease. An “early stage” was defined as an H-Y stage ≤2.5, and an “advanced stage” was defined as an H-Y stage >2.5. (4) The Wearing-off Questionnaire-9 (WOQ-9) was used to assess the presence of wearing-off (WO) in patients. (5) The Non-Motor Symptoms Scale (NMSS) was used to evaluate non-motor symptoms. (6) The Montreal Cognitive Assessment (MoCA) and Mini-Mental State Examination (MMSE) were used to assess patients’ cognitive function. (7) The 14-item version of the Hamilton Anxiety Rating Scale (HAMA-14) and the 17-item version of the Hamilton Depression Rating Scale (HAMD-17) were used to assess anxiety and depression.

### 2.5 Evaluation of motor complications

Patients were classified into PWP-MC if they exhibited at least one of the following: WO, dyskinesia, or brittle levodopa response (BR), as confirmed by movement disorder specialists. Physicians specializing in movement disorders assessed the patients for movement symptom fluctuations (WO being the most common; therefore, this study explicitly included patients with WO as the research subjects), dyskinesias, and the presence of BR. The specific types were analyzed and confirmed for accuracy by the chief physician of the Parkinson’s disease specialty clinic.

### 2.6 Laboratory testing

Fasting venous blood samples were collected from 8:00 to 11:00 am. A total of 8 mL of blood was drawn from the elbow vein and centrifuged to obtain serum for analysis using the chemiluminescence method. The levels of six serum hormones (follicle-stimulating hormone, luteinizing hormone, prolactin, progesterone, estradiol, and testosterone) were measured. Additionally, seven serum indicators of thyroid function were measured, including triiodothyronine (T3), thyroxine (T4), free triiodothyronine (FT3), FT4, thyroid-stimulating hormone (TSH), thyroglobulin antibodies (TGAb), and thyroid peroxidase antibodies (TPOAbs).

### 2.7 Other observation and recording indicators

(1) Onset delay: On the basis of the initially effective dose of LD, the drug takes effect 1 h or more later, combined with the UPDRS-III motor function score and H-Y staging.

(2) Using the UPDRS-III and UPDRS-IV, the occurrence time and duration (y), LD onset time (min), and maintenance time (min) of symptom fluctuations (WO, onset delay) were recorded, as were the occurrence time and duration (y), single LD dosage, and occurrence of BR with a single LD dosage in patients with dyskinesia. BR was defined as when the single LD dosage is 100 mg or less, resulting in disabling LID, and the disability score for dyskinesia was based on Item 33 of the UPDRS-IV (reaching at least moderate disability, ≥2 points).

(3) LEDD calculation (26): A neurologist from the research team recorded the medications used by the PD patients, including the name, dosage form, dosage, and frequency of administration, and calculated the LEDD (LEDD = LD immediate-release tablets × 1 + LD controlled-release tablets × 0.75 + Pramipexole × 100 + Amantadine × 1 + Selegiline × 10 + Pergolide prolonged-release tablets × 1).

## 3 Results

### 3.1 Demographic and clinical characteristics of PWP

Among the PWP, 37 (38.9%) had motor complications. Compared with premenopausal women with PD but without motor complications (PWP-nMC), premenopausal women with PD with motor complications (PWP-MC) had a lower age at onset but a longer disease duration and higher LEDD, HAMD, H-Y stage, UPDRS-III, and NMSS values (*p* < 0.05) (Table 1).

**TABLE 1:** Basic clinical characteristics of PWP.

Variable	Total (*N* = 95[100%])	Motor complications (−) (*N* = 58[61.1%])	Motor complications (+) (*N* = 37[38.9%])	*p*
Age, year	68.1 ± 8.9	68.8 ± 9.1	66.9 ± 8.6	0.298
Age of onset, year	63.1 ± 9.5	65.7 ± 8.9	58.9 ± 9.0	<0.001*
The course of the disease, the year	4.0 (2.0,8.0)	2.0 (1.0, 4.0)	8.0 ± 4.0	<0.001*
BMI, kg/m^2^	23.2 ± 3.1	23.6 ± 2.8	22.3 ± 3.7	0.086
Age of menarche, year	14.8 ± 1.9	14.9 ± 2.2	14.8 ± 1.6	0.734
Age of menopause, year	49.4 ± 4.5	49.6 ± 4.2	49.2 ± 4.8	0.674
Menstrual history, year	34.6 ± 4.9	34.7 ± 4.9	34.4 ± 5.0	0.804
Menstrual cycle, day	30 (28, 30)	30 (28, 30)	30 (28, 30)	0.834
Number of menstrual days	5.5 ± 1.3	5.0 (5.0, 7.0)	5.0 (4.5, 7.0)	0.746
LEDD, mg/d	468.06 ± 252.73^#^	390.05 ± 227.91^&^	579.81 ± 247.14	<0.001*
MMSE	26.0 (24.0, 28.0)	26.0 (23.0, 28.0)	25.5 ± 3.8	0.375
MoCA	20.0 ± 6.2	19.3 ± 6.7	21.0 ± 5.2	0.183
HAMA	8.0 (4.0, 13.0)	8.2 ± 7.0	10.8 ± 6.6	0.072
HAMD	8.0 (4.0, 15.0)	5.5 (3.0, 13.0)	13.32 ± 7.51	0.013*
H-Y stage	2.5 (2.0, 3.0)	2.1 ± 0.8	3.0 (2.3, 3.0)	<0.001*
H-Y stage, *n* (%)				0.003*
1	14 (14.7)	12 (20.7)	2 (5.4)	
1.5	5 (5.3)	4 (6.9)	1 (1.7)	
2	21 (22.1)	15 (25.9)	6 (16.2)	
2.5	22 (23.2)	15 (25.9)	7 (18.9)	
3	27 (28.4)	10 (17.2)	17 (45.9)	
4	6 (6.3)	2 (3.4)	4 (10.8)	
UPDRS-III	35.6 ± 17.2	31.6 ± 17.3	41.7 ± 15.3	0.005*
NMSS	58.8 ± 37.6	49.0 ± 35.8	74.2 ± 35.6	0.001*

#, *n* = 90; &, *n* = 53; *p < 0.05. Continuous variables are presented as mean ± standard deviation or median (first quartile, third quartile) based on distribution normality.

### 3.2 Comparison of serum hormone and thyroid levels in PWP with and without motor complications, WO, and dyskinesia

Compared with PWP-nMC, PWP-MC has lower levels of prolactin and T3. TSH levels were higher in PWP-MC than in PWP-nMC (*p* < 0.05). After multiple comparison corrections, only a statistically significant difference in prolactin levels was detected between the two groups (*p* < 0.0038). After adjusting for LEDD, prolactin levels remained significantly lower in PWP-MC compared to PWP-nMC (adjusted mean difference: −3.21 ng/mL, SE = 0.93; *p* = 0.008) (Table 2).

**TABLE 2:** Comparison of serum hormone and thyroid levels in PWP with or without motor complication.

Hormones	Total (*N* = 95[100%])	Motor complications			WO			Dyskinesia		
		Without	With		Without	With		Without	With	
		(−) (*N* = 58[61.1%])	(+) (*N* = 37[38.9%])	*p*	(−) (*N* = 60[63.2%])	(+) (*N* = 35[36.8%])	p	(−) (*N* = 81[85.3%])	(+) (*N* = 14[14.7%])	*p*
Luteinizing hormone	31.98 ± 14.69	32.00 ± 15.05	31.94 ± 14.32	0.987	32.07 ± 15.11	31.81 ± 14.16	0.935	31.45 ± 13.79	35.01 ± 19.46	0.406
FSH	74.96 ± 26.04	75.56 ± 26.03	74.00 ± 26.39	0.779	74.95 ± 26.14	74.96 ± 26.24	0.997	73.45 ± 24.41	83.67 ± 33.76	0.176
Prolactin^†^	5.40 (1.82, 9.33)	7.32 (3.97, 11.83)	4.63 ± 4.72	0.001*	7.35 (3.97, 11.86)	4.87 ± 4.75	0.045^#^	5.35 (3.56, 7.33)	3.17 ± 3.74	0.118
Progesterone	0.43 ± 0.28	0.39 ± 0.26	0.48 ± 0.32	0.177	0.40 ± 0.26	0.47 ± 0.32	0.267	0.41 ± 0.28	0.51 ± 0.32	0.255
Estradiol	17.70 (17.50, 27.00)/26.01 ± 25.31	17.50 (17.50, 25.25)	21.00 (17.50, 28.00)	0.389	26.69 ± 16.70	24.83 ± 12.74	0.731	25.82 ± 26.81	27.11 ± 14.41	0.861
Testosterone	33.04 ± 15.55	33.63 ± 16.30	32.09 ± 14.47	0.640	34.00 ± 16.17	31.38 ± 14.51	0.431	32.73 ± 16.17	34.81 ± 12.68	0.647
T3	1.42 ± 0.29	1.50 ± 0.30	1.37 ± 2.73	0.042*	1.52 ± 0.27	1.36 ± 0.29	0.010^#^	1.42 ± 0.28	1.45 ± 0.35	0.699
T4	102.87 ± 20.99	100.60 ± 22.21	106.44 ± 18.66	0.187	100.20 ± 22.25	107.46 ± 18.01	0.104	102.73 ± 21.31	103.67 ± 19.74	0.878
FT3	3.98 ± 6.55	3.93 ± 0.63	4.06 ± 0.69	0.333	4.17 ± 0.53	3.87 ± 0.70	0.035^#^	4.00 ± 0.61	3.95 ± 0.90	0.829
FT4	12.45 (11.33, 13.87)	12.47 (11.28, 13.81)	13.33 ± 2.56	0.412	12.49 (11.24, 13.88)	13.44 ± 2.59	0.472	12.52 (11.35, 13.67)	13.39 ± 2.25	0.694
TSH	1.86 (1.15, 2.80)	1.75 (0.92, 2.30)	2.07 (1.28, 3.01)	0.047*	1.97 (1.38, 2.52)	1.77 (0.94, 2.31)	0.265	1.93 (1.56, 2.31)	1.75 (0.98, 2.14)	0.422
TgAb	0.45 (0.45, 0.45)	0.45 (0.45, 0.64)	0.45 (0.45, 0.45)	0.651	0.45 (0.45, 0.63)	0.45 (0.45, 0.46)	0.685	0.45 (0.45, 0.65)	0.45 (0.45, 0.46)	0.937
TPOAb	0.31 (1.25, 1.00)	0.35 (0.13, 0.94)	0.13 (0.13, 1.16)	0.331	0.34 (0.12, 0.93)	0.14 (0.14, 1.17)	0.374	0.33 (0.14, 0.92)	0.19 (0.16, 1.23)	0.343

*After Bonferroni correction for multiple comparisons, statistical significance was observed (with a threshold of *p* < 0.0038 when considering 13 parameters).

^#^After Bonferroni correction for multiple comparisons, statistical significance was observed (with a threshold of *p* < 0.0036 when considering 13 parameters).

^†^Prolactin difference persists after LEDD adjustment (β = −3.21, SE = 0.93, *p* = 0.008).

The prolactin, T3, and FT3 levels were lower in the PWP with WO than in the PWP without WO (*p* < 0.05). After adjusting for multiple comparisons, there was no statistically significant difference between the two groups (*p* > 0.05) (Table 2).

There were no statistically significant differences in the levels of sex hormones or thyroid hormones between PWP with dyskinesia and PWP without dyskinesia (*p* > 0.05) (Table 2).

### 3.3 Comparison of clinical characteristics between PWP with and without WO

As shown in Table 3, 35 patients (36.8%) presented with WO among the PWP. Compared with PWP without WO, PWP with WO had a younger age at onset but had a longer disease duration, higher LEDD, higher H-Y stage, higher UPDRS-III score, and higher HAMA, HAMD, and NMSS scores (*p* < 0.05).

**TABLE 3:** Comparison of clinical characteristics between PWP with and without WO.

Variable	Total (*N* = 95[100%])	WO (−) (*N* = 60[63.2%])	WO (+) (*N* = 35[36.8%])	*p*
Age, year	68.1 ± 8.9	68.8 ± 8.9	66.9 ± 8.8	0.317
Age of onset, year	63.1 ± 9.5	65.5 ± 8.9	58.8 ± 9.1	0.001*
The course of the disease, the year	4.0 (2.0,8.0)	2.0 (1.0, 4.0)	8.0 ± 4.1	0.000*
BMI, kg/m^2^	23.2 ± 3.1	23.4 ± 2.9	22.5 ± 3.7	0.157
Age of menarche, year	14.8 ± 1.9	14.9 ± 2.1	14.7 ± 1.6	0.627
Age of menopause, year	49.4 ± 4.5	49.3 ± 4.5	49.7 ± 4.4	0.709
Menstrual cycle, day	30 28, 30)	30 (28, 30)	30 (28,30)	0.943
Number of menstrual days	5.5 ± 1.3	5.5 ± 1.3	5.5 ± 1.4	0.880
Menstrual history, year	34.6 ± 4.9	34.4 ± 5.2	34.9 ± 4.4	0.594
LEDD, mg/d	468.06 ± 252.73^#^	395.41 ± 226.93^&^	582.23 ± 251.89	0.000*
MMSE	24.7 ± 5.2	24.1 ± 5.8	25.6 ± 3.7	0.161
MoCA	20.0 ± 6.2	19.3 ± 6.8	21.1 ± 4.9	0.169
HAMA	9.2 ± 7.0	8.0 ± 7.0	11.4 ± 6.5	0.021*
HAMD	10.1 ± 7.7	8.5 ± 7.5	12.9 ± 7.3	0.007*
H-Y stage	2.4 ± 0.8	2.2 ± 0.8	2.7 ± 0.7	0.002*
UPDRS-III	35.6 ± 17.2	32.0 ± 17.2	41.6 ± 15.6	0.008*
NMSS	58.8 ± 37.6	48.5 ± 35.3	76.5 ± 35.2	0.000*

^#^*n* = 90; ^&^*n* = 55; *p < 0.05. Data expressed as median (Q1, Q3) indicate non-normally distributed variables.

### 3.4 Binary logistic regression analysis of motor complications and WO in PWP

As shown in Table 4, the significant factors from the comparisons between the subgroup with motor complications and that with non-motor complications in Table 1 and Table 2 included age at onset, disease duration, LEDD, H-Y stage, HAMD, UPDRS-III and NMSS scores, and prolactin, T3, and TSH levels, which were used as independent variables. In contrast, motor complications were used as the dependent variable in the multivariate logistic regression analysis. The results of the multivariate logistic regression analysis revealed that prolactin, T3, and TSH levels were not significantly different between the groups (*p* > 0.05). An older age at onset was a protective factor against motor complications in PWP, whereas a longer disease duration and higher NMSS score were identified as risk factors for motor complications in PWP.

**TABLE 4:** Binary logistic regression analysis of motor complications and WO in PWP.

Variable	Motor complication						WO					
	B	SE	Wald χ*2*	*P*	OR	95%CI	B	SE	Wald χ*2*	*P*	OR	95%CI
Constant	2.868	2.497	1.320	0.251	17.600		−4.934	3.861	1.633	0.201	0.007	
NMS Non-Motor Symptoms Scale	0.019	0.008	5.244	0.022*	1.019	1.003 1.036	0.023	0.011	4.933	0.026*	1.024	1.003 1.036
Age of onset	−0.100	0.041	6.016	0.014*	0.905	0.836 0.980	−0.082	0.041	4.094	0.043*	0.921	0.850 0.997
Disease duration	0.377	0.102	13.586	<0.001*	1.458	1.193 1.782	0.308	0.119	6.693	0.010*	1.360	1.077 1.717

*p < 0.05

As shown in Table 2 and Table 3, factors with statistical significance in the comparison between the WO and non-WO subgroups, including age at onset, disease duration, LEDD, H-Y stage, UPDRS-III, HAMA, HAMD, and NMSS scores, and prolactin, T3, and FT3 levels, were used as independent variables, with WO used as the dependent variable in the multivariate logistic regression analysis (Table 4). The results revealed that prolactin, T3, and TSH levels were not significantly different according to the multivariate logistic regression analysis (*p* > 0.05). An older age at onset was a protective factor against WO in PWP, whereas a longer disease duration and higher NMSS score were identified as risk factors for WO in PWP.

### 3.5 Correlations of serum hormone levels, thyroid hormone levels, and menstrual factors with clinical characteristics in PWP

As shown in Table 5, we analyzed the correlations between the levels of serum hormones (including serum luteinizing hormone, follicle-stimulating hormone, prolactin, progesterone, estradiol, and testosterone) in PWP and motor function according to the UPDRS-III, H-Y stage, and MMSE, MoCA, HAMD, HAMA, and NMSS scores. The results revealed a negative correlation between serum estradiol levels in PWP and HAMA scores (r = −0.208, *p* = 0.043), indicating that higher estradiol levels in PWP were associated with lower HAMA scores.

**TABLE 5:** The correlation study of serum hormone levels, thyroid function levels, and menstrual factors with clinical characteristics in PWP.

Variable	UPDRS-III	H-Y stage	MMSE	MoCA	HAMD	HAMA	NMSS
Luteinizing hormone	0.06 (0.52)	0.004 (0.97)	0.003 (0.976)	0.045 (0.663)	0.006 (0.952)	0.071 (0.496)	−0.038 (0.715)
FSH	−0.059 (0.568)	−0.061 (0.555)	−0.01 (0.924)	0.031 (0.764)	0.107 (0.304)	0.133 (0.200)	−0.063 (0.547)
Prolactin	−0.018 (0.859)	−0.082 (0.431)	−0.038 (0.717)	−0.055 (0.594)	0.053 (0.609)	0.069 (0.507)	−0.045 (0.663)
Progesterone	0.015 (0.886)	0.108 (0.296)	−0.083 (0.422)	−0.028 (0.788)	−0.057 (0.586)	0.137 (0.185)	0.033 (0.750)
Estradiol	0.171 (0.098)	0.164 (0.113)	0.150 (0.148)	−0.002 (0.987)	−0.138 (0.183)	−0.208 (0.043)	0.088 (0.399)
Testosterone	−0.115 (0.266)	0.091 (0.380)	0.002 (0.988)	−0.021 (0.842)	−0.033 (0.751)	−0.002 (0.983)	−0.017 (0.870)
T3	−0.163 (0.113)	−0.092 (0.374)	0.129 (0.213)	0.097 (0.348)	0.000 (0.997)	0.044 (0.671)	0.009 (0.932)
T4	−0.04 (0.699)	−0.005 (0.960)	−0.045 (0.665)	0.104 (0.317)	−0.012 (0.909)	−0.016 (0.876)	0.084 (0.416)
FT3	−0.173 (0.093)	−0.141 (0.172)	0.171 (0.097)	0.103 (0.320)	−0.016 (0.877)	0.017 (0.872)	0.55 (0.598)
FT4	−0.138 (0.183)	−0.108 (0.296)	0.142 (0.169)	−0.076 (0.461)	−0.01 (0.921)	−0.002 (0.988)	0.205 (0.046)
TSH	0.002 (0.988)	−0.004 (0.968)	−0.136 (0.189)	−0.218 (0.033)	0.090 (0.386)	0.222 (0.031)	−0.08 (0.441)
TgAb	0.077 (0.458)	0.011 (0.916)	0.070 (0.501)	−0.067 (0.517)	0.031 (0.766)	0.030 (0.770)	0.124 (0.230)
TPOAb	0.076 (0.465)	−0.004 (0.971)	0.012 (0.908)	−0.035 (0.737)	−0.015 (0.882)	0.053 (0.609)	−0.070 (0.499)
Age of menarche, year	0.107 (0.303)	0.050 (0.632)	−0.264 (0.01)	−0.297 (0.004)	−0.001 (0.996)	0.035 (0.738)	−0.001 (0.995)
Age of menopause, year	−0.136 (0.188)	−0.115 (0.267)	0.064 (0.540)	0.144 (0.163)	0.040 (0.706)	−0.012 (0.909)	0.016 (0.874)
Total menstrual period	−0.166 (0.107)	−0.158 (0.126)	0.198 (0.058)	0.278 (0.006)	0.033 (0.750)	−0.036 (0.732)	0.015 (0.884)
Menstrual cycle	−0.246 (0.016)	−0.236 (0.021)	−0.110 (0.288)	−0.017 (0.866)	0.105 (0.313)	0.179 (0.082)	0.043 (0.682)
Menstrual days	0.018 (0.865)	−0.026 (0.805)	−0.026 (0.803)	−0.014 (0.894)	0.187 (0.069)	0.215 (0.036)	0.214 (0.037)

The variable is represented as r (*p*-value).

In this study, we analyzed the correlations between serum thyroid function indicators, including T3, T4, FT3, FT4, TSH, TGAb, and TPOAb levels, and PD patient assessment results, including the H-Y stage and UPDRS-III, MMSE, MoCA, HAMD, HAMA, and NMSS scores. The results revealed a positive correlation between FT4 levels in PWP and NMSS scores (*r* = 0.205, *p* = 0.046), a positive correlation between TSH levels and HAMA scores (*r* = 0.222, *p* = 0.031), and a negative correlation between TSH levels and MoCA scores (*r* = −0.218, *p* = 0.033). The results suggest that higher FT4 levels are associated with more severe non-motor symptoms in PWP, and higher TSH levels are linked to increased anxiety symptoms and cognitive impairment in these patients.

We also analyzed menstrual factors, including age at menarche, age at menopause, total menstrual duration, menstrual cycle duration, and menstrual duration, and their correlations with the clinical characteristics of PWP. The results revealed a negative correlation between age at menarche in PWP and MMSE (*r* = −0.264, *p* = 0.01) and MoCA scores (*r* = −0.297, *p* = 0.004), indicating that an older age at menarche was associated with more severe cognitive impairment in PWP. The total menstrual duration was positively correlated with MoCA scores (*r* = 0.278, *p* = 0.006), suggesting that a more extended period was associated with better MoCA performance and less cognitive decline. Menstrual cycle duration was negatively correlated with the UPDRS-III score (*r* = −0.246, *p* = 0.016) and H-Y stage (*r* = −0.236, *p* = 0.021), indicating that a longer menstrual cycle was associated with milder motor symptoms and a lower H-Y stage. The number of menstrual days was positively correlated with HAMA (*r* = 0.215, *p* = 0.036) and NMSS scores (*r* = 0.214, *p* = 0.037), suggesting that a greater number of menstrual days was associated with increased anxiety and non-motor symptoms in PWP.

### 3.6 Correlations between serum hormone levels, thyroid hormone levels, and menstrual factors and motor complications in PWP

As shown in Table 6, an analysis was conducted on the correlation between serum sex hormone levels and motor complications in PWP. The results revealed no correlation between sex hormone levels in PWP and various scale scores (*p* > 0.05).

**TABLE 6:** The correlation between serum hormone levels, thyroid hormone levels, menstrual factors, and motor complications in PWP.

Variable	UPDRS-III	H-Y stage	MMSE	MoCA	HAMD	HAMA	NMSS
Luteinizing hormone	0.129 (0.448)	0.074 (0.662)	−0.107 (0.527)	−0.050 (0.767)	−0.074 (0.664)	−0.005 (0.997)	−0.083 (0.627)
FSH	0.082 (0.629)	0.041 (0.808)	−0.071 (0.676)	0.000 (0.998)	0.155 (0.361)	0.219 (0.193)	0.032 (0.850)
Prolactin	0.067 (0.694)	−0.027 (0.874)	0.052 (0.758)	−0.079 (0.643)	0.067 (0.692)	0.021 (0.900)	−0.001 (0.997)
Progesterone	−0.219 (0.192)	−0.031 (0.856)	−0.04 (0.816)	0.089 (0.602)	−0.210 (0.211)	−0.029 (0.865)	−0.26 (0.120)
Estradiol	0.085 (0.618)	−0.09 (0.594)	0.172 (0.308)	0.055 (0.746)	−0.169 (0.307)	−0.307 (0.065)	0.002 (0.990)
Testosterone	0.222 (0.186)	−0.096 (0.574)	0.097 (0.569)	0.122 (0.472)	−0.287 (0.085)	−0.186 (0.271)	−0.148 (0.382)
T3	−0.344 (0.037)	−0.450 (0.005)	0.259 (0.122)	0.331 (0.045)	−0.058 (0.734)	0.114 (0.501)	−0.101 (0.553)
T4	−0.406 (0.013)	−0.209 (0.214)	−0.113 (0.506)	−0.164 (0.332)	−0.043 (0.802)	0.021 (0.903)	0.023 (0.891)
FT3	−0.319 (0.054)	−0.476 (0.003)	0.393 (0.016)	0.381 (0.020)	−0.027 (0.874)	0.113 (0.507)	−0.035 (0.836)
FT4	−0.422 (0.009)	−0.365 (0.026)	−0.314 (0.058)	−0.261 (0.119)	−0.017 (0.918)	0.228 (0.176)	0.037 (0.830)
TSH	−0.015 (0.930)	−0.106 (0.532)	−0.201 (0.232)	−0.209 (0.215)	0.085 (0.615)	0.160 (0.343)	0.051 (0.766)
TgAb	0.095 (0.575)	0.052 (0.758)	0.083 (0.625)	−0.057 (0.739)	−0.052 (0.759)	−0.096 (0.573)	−0.013 (0.939)
TPOAb	0.033 (0.845)	0.024 (0.890)	0.189 (0.262)	0.118 (0.487)	0.014 (0.933)	0.035 (0.837)	−0.022 (0.899)
Age of menarche, year	−0.160 (0.343)	−0.048 (0.776)	−0.223 (0.184)	−0.356 (0.030)	−0.028 (0.867)	−0.083 (0.624)	0.047 (0.780)
Age of menopause, year	−0.003 (0.984)	0.015 (0.930)	0.009 (0.956)	−0.084 (0.623)	0.335 (0.043)	0.258 (0.123)	0.237 (0.158)
Total menstrual period	0.047 (0.781)	0.011 (0.947)	0.080 (0.639)	0.194 (0.250)	0.352 (0.033)	0.278 (0.096)	0.215 (0.201)
Menstrual cycle	−0.362 (0.028)	−0.278 (0.096)	−0.228 (0.174)	−0.157 (0.353)	0.329 (0.047)	0.451 (0.005)	0.287 (0.084)
Menstrual days	0.051 (0.763)	0.075 (0.665)	0.084 (0.621)	0.110 (0.517)	0.275 (0.099)	0.238 (0.157)	0.325 (0.050)

The variable is represented as r (*p*-value).

We also analyzed the correlation between thyroid function and motor complications in PWP. The results revealed that there was a negative correlation between T3 levels and the UPDRS-III score (*r* = −0.344, *p* = 0.037) and H-Y stage (*r* = −0.450, *p* = 0.005) and a positive correlation with the MoCA score (*r* = 0.331, *p* = 0.045). These results suggest that higher T3 levels are associated with milder motor symptoms, lower H-Y stages, and better cognitive performance, as assessed by the MoCA. Similarly, T4 levels were negatively correlated with the UPDRS-III score (*r* = −0.406, *p* = 0.013), indicating that higher T4 levels were associated with milder motor symptoms. FT3 levels were negatively correlated with the H-Y stage (*r* = −0.476, *p* = 0.003) and positively correlated with the MMSE score (*r* = 0.393, *p* = 0.016) and MoCA score (*r* = 0.381, *p* = 0.020), suggesting that higher FT3 levels were associated with a lower H-Y stage and better cognitive performance, as assessed by the MMSE and MoCA. FT4 levels were negatively correlated with the UPDRS-III score (*r* = −0.422, *p* = 0.009) and H-Y stage (*r* = −0.365, *p* = 0.026), indicating that higher FT4 levels were associated with milder motor symptoms and a lower H-Y stage.

Furthermore, we analyzed the correlation between menstrual factors and motor complications in PWP. The results revealed a positive correlation between age at menopause and the HAMD score (*r* = 0.335, *p* = 0.043), indicating that an older age at menopause was associated with more severe depressive symptoms in the PWP-MC. The total menstrual duration was also positively correlated with the HAMD score (*r* = 0.352, *p* = 0.033), indicating that a longer total menstrual duration was associated with more severe depressive symptoms. The menstrual cycle was negatively correlated with the UPDRS-III score (*r* = −0.362, *p* = 0.028) and positively correlated with the HAMD score (*r* = 0.329, *p* = 0.047) and HAMA score (*r* = 0.451, *p* = 0.005), suggesting that a longer total menstrual duration was associated with milder motor symptoms but more severe anxiety and depressive symptoms in PWP-MC.

### 3.7 Correlations among sex hormones, thyroid function, and menstrual factors in PWP-nMC

As shown in Table 7, the analysis of the correlations between sex hormone levels and the characteristics of PWP-nMC revealed that there was no correlation between sex hormones and the clinical characteristics of or disease severity in PWP-nMC (*p* > 0.05).

**TABLE 7:** The correlation between sex hormones, thyroid function, menstrual factors, and clinical characteristics in PWP-nMC.

Variable	UPDRS-III	H-Y stage	MMSE	MoCA	HAMD	HAMA	NMSS
Luteinizing hormone	0.029 (0.829)	−0.020 (0.879)	0.079 (0.554)	0.091 (0.498)	0.066 (0.662)	0.016 (0.906)	−0.014 (0.919)
FSH	−0.130 (0.332)	−0.075 (0.576)	0.096 (0.474)	−0.053 (0.690)	0.154 (0.250)	0.065 (0.626)	−0.112 (0.401)
Prolactin	0.105 (0.435)	0.077 (0.566)	−0.006 (0.963)	−0.032 (0.813)	0.169 (0.204)	0.171 (0.199)	0.119 (0.374)
Progesterone	0.108 (0.420)	0.154 (0.247)	−0.122 (0.362)	−0.132 (0.323)	0.121 (0.365)	0.250 (0.059)	0.181 (0.173)
Estradiol	0.185 (0.165)	0.235 (0.076)	0.111 (0.405)	−0.044 (0.742)	−0.193 (0.146)	−0.203 (0.126)	0.047 (0.728)
Testosterone	−0.048 (0.718)	0.241 (0.068)	0.012 (0.930)	−0.074 (0.583)	0.086 (0.523)	0.048 (0.720)	0.081 (0.547)
T3	−0.176 (0.186)	−0.111 (0.405)	0.160 (0.230)	0.075 (0.575)	−0.061 (0.652)	−0.042 (0.754)	−0.040 (0.767)
T4	0.067 (0.616)	0.037 (0.785)	0.051 (0.704)	−0.024 (0.858)	−0.078 (0.562)	−0.031 (0.817)	0.053 (0.694)
FT3	−0.149 (0.264)	−0.072 (0.589)	0.111 (0.408)	0.011 (0.934)	−0.030 (0.824)	−0.084 (0.531)	0.064 (0.635)
FT4	−0.016 (0.905)	−0.012 (0.927)	−0.079 (0.554)	0.012 (0.930)	−0.042 (0.753)	−0.075 (0.577)	0.268 (0.042)
TSH	0.064 (0.633)	0.159 (0.232)	0.108 (0.419)	−0.188 (0.158)	0.181 (0.173)	0.374 (0.004)	−0.047 (0.724)
TgAb	0.096 (0.476)	0.032 (0.810)	0.052 (0.700)	−0.059 (0.662)	0.101 (0.452)	0.129 (0.334)	0.226 (0.089)
TPOAb	0.161 (0.227)	0.041 (0.763)	−0.095 (0.477)	−0.120 (0.370)	0.038 (0.778)	0.105 (0.434)	−0.008 (0.952)
Age of menarche, year	0.238 (0.072)	0.147 (0.230)	−0.332 (0.011)	−0.296 (0.024)	0.095 (0.477)	0.160 (0.231)	−0.005 (0.972)
Age of menopause, year	−0.211 (0.112)	−0.148 (0.267)	0.114 (0.396)	0.239 (0.071)	−0.125 (0.348)	−0.143 (0.283)	−0.114 (0.396)
Total menstrual period	−0.287 (0.029)	−0.235 (0.076)	0.316 (0.016)	0.337 (0.010)	−0.133 (0.321)	−0.194 (0.144)	−0.096 (0.472)
Menstrual cycle	−0.177 (0.183)	−0.223 (0.092)	−0.019 (0.887)	0.043 (0.748)	−0.014 (0.919)	0.023 (0.865)	−0.053 (0.693)
Menstrual days	−0.017 (0.899)	−0.100 (0.456)	−0.131 (0.328)	−0.083 (0.583)	0.140 (0.293)	0.224 (0.0)91)	0.140 (0.294)

The variable is represented as r (*p-*value). PWP-nMC: Postmenopausal women with PD without motor complications (i.e., no WO, dyskinesia, or BR).

We further analyzed the correlation between thyroid function and the characteristics of PWP-nMC. The results indicated a positive correlation between FT4 levels and NMSS scores (*r* = 0.268, *p* = 0.042), suggesting that higher FT4 levels in PWP were associated with more severe non-motor symptoms. Additionally, there was a positive correlation between TSH levels and HAMA scores (*r* = 0.374, *p* = 0.004), indicating that higher TSH levels were associated with more severe anxiety symptoms in patients.

(1) Moreover, the analysis of menstrual factors and the characteristics of PWP-nMC revealed a negative correlation between age at menarche and MMSE (*r* = −0.332, *p* = 0.011) and MoCA (*r* = −0.296, *p* = 0.024) scores, suggesting that an older age at menarche was associated with poorer cognitive performance, as assessed by the MMSE and MoCA. The total menstrual duration was negatively correlated with the UPDRS-III score (*r* = −0.287, *p* = 0.029) and positively correlated with the MMSE (*r* = 0.316, *p* = 0.016) and MoCA (*r* = 0.337, *p* = 0.010) scores, indicating that a longer menstrual duration was associated with milder motor symptoms and better cognitive performance, as assessed by the MMSE and MoCA.

## 4 Discussion

Our previous research revealed that premenopausal women experience exacerbated PD symptoms during menstruation (27). In this study, further analysis of the effects of menstrual factors on PWP revealed that an older age at onset is inversely associated with motor-related complications in PWP. Moreover, a longer disease duration and higher NMSS score are risk factors for motor-related complications in PWP. The length of time for which sex hormones provide protection is closely related to these findings, as PWP have the lowest estrogen levels, which may impact dopamine pathways in the midbrain and cortex during the menstrual period, leading to indirect dopamine reduction (18). Numerous studies have confirmed the critical role of sex hormones in the pathogenesis of PD, especially the significant influence of estrogen on the survival and development of neurons (18). This finding is consistent with the clinical observation that early menopause is associated with disease progression in PWP. Beyond hormonal pathways, the inverse relationship between advanced onset age and motor complications reflects delayed levodopa initiation and reduced cumulative dopaminergic exposure. Older-onset patients (mean: 65.7 ± 8.9 years) typically commence treatment later than younger counterparts, yielding: Shorter duration of pulsatile dopaminergic stimulation; Attenuated maladaptive neuroplasticity in corticostriatal circuits; Lower incidence of LID. Conversely, younger patients undergoing prolonged dopaminergic therapy exhibit accelerated synaptic remodeling and heightened LID susceptibility.

PWP-MC have lower prolactin levels than PWP-nMC. Since PD is a neurodegenerative disease characterized by dopaminergic neuron loss, the dopaminergic system is interconnected with the hypothalamic–pituitary–thyroid axis. Dopamine (DA) upregulates thyrotropin-releasing hormone (TRH) release while downregulating TSH and thyroid hormone levels (28, 29). DA can regulate prolactin synthesis through two pathways: directly inhibiting pituitary prolactin synthesis in the neuroendocrine system and indirectly stimulating prolactin synthesis by upregulating TRH and affecting prolactin-producing cells in the pituitary gland (19, 28, 29). The H-Y stage was higher in PWP-MC than in PWP-nMC (*p* < 0.005), indicating a more severe disease state in the former group, possibly due to more prominent dopaminergic neuron damage and significant DA reduction. Combined with the lower prolactin levels in PWP-MC, the stimulatory effect of DA on TRH to stimulate pituitary lactotroph cells to produce prolactin is more vital than its direct inhibitory effect on pituitary prolactin synthesis in PWP-MC, contrary to the conventional belief about the role of DA in the neuroendocrine system (28, 29). Although there are differences in prolactin levels between PWP with and without motor-related complications, logistic regression analysis did not reveal statistical significance, indicating that prolactin is not an independent factor influencing motor-related complications in PWP.

Correlation analysis of sex hormone levels with various scale scores in PWP revealed only a trend toward a negative correlation between estradiol levels and HAMA scores, which was not statistically significant, suggesting a possible association between low estrogen levels and anxiety symptoms in PWP. This finding indirectly indicates that the decrease in estrogen levels is related to the onset of PD, but its relationship with the occurrence of motor-related complications is still unclear (30). Further analysis revealed that other sex hormone indicators did not correlate with the clinical phenotypes of motor-related complications in PWP. Additionally, there was no correlation between sex hormone levels and various scale scores in the group of PWP-MC. These findings suggest that sex hormones are not significantly related to the disease condition in PWP, which may be attributed to the low and stable estrogen levels in these patients (29, 31).

This study analyzed the correlations among thyroid function, clinical features, and PD severity. We found that: ➀ Lower levels of T3 are associated with more severe motor symptoms, higher H-Y stages, and poorer cognitive function in female PD patients; ➁ lower levels of T4 are associated with more severe motor symptoms in PD patients; ➂ lower levels of FT3 are associated with higher H-Y stages and poorer cognitive function; ➃ lower levels of FT4 are associated with more severe motor symptoms and higher H-Y stages in PD patients; and ➄ higher levels of TSH are associated with more severe cognitive dysfunction. These findings suggest that lower thyroid function is associated with more severe motor and non-motor symptoms in female PD patients. Previous studies on the correlation between thyroid function and PD are limited. Research by Tadashi Umehara et al. revealed that TH levels, especially FT3 levels, were negatively correlated with motor function in newly diagnosed PD patients, but the relationship with motor complications was not addressed (32). Our study revealed a negative correlation between FT3 levels and H-Y stage in PD patients with motor complications. The results of this study are similar to those of the study by Tadashi Umehara, who reported more severe symptoms, motor complications, and dyskinesias in PWP, suggesting that lower FT3 levels are associated with more severe motor impairments.

Additionally, we found that both FT4 and FT3 levels were negatively correlated with the UPDRS-III score and H-Y stage. In contrast, thyroid hormone levels were negatively correlated with the H-Y stage, indicating a consistent effect of thyroid hormone levels on motor symptoms and disease severity in PWP-MC (mainly those with dyskinesias). FT3 and T3 levels were positively correlated with the characteristics of PWP-MC, possibly because patients with higher FT3 and T3 levels had less severe disease. For PWP, this study revealed a negative correlation between TSH levels and MoCA scores, indicating a negative correlation between TSH levels and cognitive function. Previous studies have shown a negative correlation between local cerebral blood flow and serum TSH levels in senile dementia patients with normal thyroid function, suggesting that changes in TSH levels within the normal range are related to regional cerebral blood flow, affecting cognitive function. Elevated TSH levels in PWP may decrease local cerebral blood flow, resulting in decreased cognitive function in these patients (33).

Furthermore, we found that FT4 levels in PWP were positively correlated with non-motor symptoms and that TSH levels were positively correlated with anxiety symptoms; in PWP-nMC, TSH levels were positively correlated with anxiety symptoms. Considering that the clinical manifestations of thyroid dysfunction (hypothyroidism or hyperthyroidism), such as apathy, delayed reactions, fatigue, emaciation, depression, anxiety, and other symptoms, are similar to the non-motor symptoms of PD, it is sometimes difficult to differentiate symptoms accurately in clinical practice, and these correlations may be due to concomitant thyroid dysfunction in PD patients. This finding is similar to those of previous studies (24). This study expands the understanding of the correlation between thyroid function and motor and non-motor symptoms in female PD patients.

Previous studies have analyzed the correlation between estrogen and PD, with most suggesting a protective role of estrogen in PD patients (31, 34), but there are also different views (35). Since estrogen levels in women of childbearing age are influenced by menstrual cycle fluctuations, a single measurement of sex hormone levels cannot represent overall levels.

In this study, PWP were included, their estrogen levels were measured, and menstrual cycle factors were investigated. The correlations between menstrual cycle factors and the characteristics of PWP-MC and PWP-nMC were analyzed. In PWP, age at menarche was negatively correlated with cognitive function, total menstrual duration was positively associated with cognitive function, menstrual cycle duration was negatively correlated with motor symptoms and disease severity, and the number of menstrual days were positively correlated with anxiety symptoms and non-motor symptoms. Overall, the longer the total menstrual or menstrual cycle duration was, the milder the motor symptoms, non-motor symptoms, and disease severity were in postmenopausal female PD patients, similar to the findings of previous studies (18, 36).

For the PWP-MC, age at menarche was negatively correlated with cognitive function, and total menstrual duration was negatively correlated with motor symptoms and positively correlated with cognitive function. This group exhibited a pattern in which longer exposure to sex hormones (longer total menstrual duration) was associated with milder motor symptoms and mental impairments, which is different from the findings in male PD patients. For the PWP-MC, total menstrual duration was positively correlated with depressive symptoms; menstrual cycle duration was negatively correlated with motor symptoms and positively correlated with anxiety and depression; the number of menstrual days was positively correlated with non-motor symptoms; and age at menopause was positively correlated with depressive symptoms. Therefore, the longer the total menstrual duration is, the milder a patient’s motor symptoms and non-motor symptoms are, but the more severe their anxiety and depression.

This study systematically investigated thyroid function, sex hormones, and menstrual cycle factors in PWP for the first time and compared the characteristics of thyroid hormone levels, sex hormone levels, and menstrual cycle factors in PWP with and without motor complications. Logistic regression analysis was used to examine the relationships between thyroid function, sex hormone levels, and menstrual cycle factors and the occurrence of motor complications in PWP. The results revealed that in PWP-MC, lower L3, T4, FT3, and FT4 levels were associated with more severe motor symptoms, higher H-Y stages, and poorer cognitive function, whereas TSH levels were related to cognitive function. This study analyzed the correlations between clinical features and disease severity in PD patients and sex hormone levels, thyroid function, and menstrual cycle factors in PWP-MC and PWP-nMC for the first time.

While our study establishes correlational associations, we propose four literature-supported mechanistic pathways bridging hormonal alterations and motor complications: (1) Thyroid hormone dysregulation, where reduced T3/FT3 levels in motor complications patients (H-Y stage correlation: *r* = −0.476, *p* = 0.003) exacerbate dyskinesia via mitochondrial dysfunction (impaired Complex I activity) and striatal D1 receptor hypersensitivity–consistent with rodent data showing 40% increased LID severity in hypothyroid models reversible by T3 supplementation; (2) Prolactin biphasic regulation, with lower levels reflecting both levodopa-mediated D2 agonism and hypothalamic-pituitary-thyroid (HPT) axis disruption, wherein nigrostriatal degeneration impairs thyrotropin-releasing hormone (TRH)-dependent prolactin synthesis (*p* < 0.005 for advanced H-Y stages); (3) Estrogen-mediated neuroprotection, evidenced by attenuated motor symptoms correlating with prolonged reproductive lifespan (*r* = −0.246, *p* = 0.016), potentially through glial cell line-derived neurotrophic factor (GDNF)-enhanced synaptic stability countering pulsatile dopamine-induced plasticity; and (4) HPT-striatal pathway activation, where TRH overexpression in dyskinetic rats drives mTOR-dependent synaptic remodeling, explaining the 70% LID reduction with mTOR inhibitors (e.g., rapamycin) in PD models. To translate these correlations into causal evidence, we recommend: (i) longitudinal profiling of hormone dynamics pre-/post-levodopa dosing, (ii) stratified assessment of striatal mTOR activation in postmortem tissues by thyroid function, and (iii) targeted hormone replacement trials in established PD models.

There are several limitations to this study. First, for the study of thyroid hormone levels, sex hormone levels, and menstrual cycle factors in PWP, a standard control group was not included, so differences between the study population and the average population cannot be deduced. Second, fertile female PD patients were excluded, as studies have shown that this group often experiences worsening symptoms during menstruation, suggesting that sex hormone levels may influence this group more and that exploring the correlation between sex hormone levels and clinical characteristics in this group has clinical significance. Third, while beyond our scope, future studies should include matched male PD cohorts with equivalent disease severity and LEDD to isolate gender-specific mechanisms. For instance, comparing prolactin dynamics in males with analogous motor complications could clarify whether observed effects require ovarian hormone interactions.

## Data Availability

The original contributions presented in this study are included in this article/supplementary material, further inquiries can be directed to the corresponding author.
